# Case Report: Role of Ketone Monitoring in Diabetic Ketoacidosis With Acute Kidney Injury: Better Safe Than Sorry

**DOI:** 10.3389/fped.2022.869299

**Published:** 2022-05-06

**Authors:** Davide Tinti, Silvia Savastio, Licia Peruzzi, Luisa De Sanctis, Ivana Rabbone

**Affiliations:** ^1^Department of Pediatrics, University of Turin, Turin, Italy; ^2^Division of Pediatrics, Department of Health Science, University of Piemonte Orientale, Novara, Italy; ^3^Pediatric Nephrology Unit, Città della Salute e della Scienza di Torino, Turin, Italy

**Keywords:** type 1 diabetes (T1D), diabetic ketoacidosis, ketones, acute kidney injury (AKI), case series

## Abstract

**Background:**

Type 1 Diabetes (T1D) is a well-known endocrinological disease in children and adolescents that is characterized by immune-mediated destruction of pancreatic β-cells, leading to partial or total insulin deficiency, with an onset that can be subtle (polydipsia, polyuria, weight loss) or abrupt (Diabetic Keto-Acidosis, hereafter DKA, or, although rarely, Hyperosmolar Hyperglycemic State, hereafter HHS). Severe DKA risk at the onset of T1D has recently significantly increased during the SARS-CoV-2 pandemic with life-threatening complications often due to its management. DKA is marked by low pH (<7.3) and bicarbonates (<15 mmol/L) in the presence of ketone bodies in plasma or urine, while HHS has normal pH (>7.3) and bicarbonates (>15 mmol/L) with no or very low ketone bodies. Despite this, ketone monitoring is not universally available, and DKA diagnosis is mainly based on pH and bicarbonates. A proper diagnosis of the right form with main elements (pH, bicarbonates, ketones) is essential to begin the right treatment and to identify organ damage (such as acute kidney injury).

**Case Presentations:**

In this series, we describe 3 case reports in which the onset of T1D was abrupt with severe acidosis (pH < 7.1) in the absence of both DKA and HHS. In a further evaluation, all 3 patients showed acute kidney injury, which caused low bicarbonates and severe acidosis without increasing ketone bodies.

**Conclusion:**

Even if it is not routinely recommended, a proper treatment that included bicarbonates was then started, with a good response in terms of clinical and laboratory values. With this case series, we would like to encourage emergency physicians to monitor ketones, which are diriment for a proper diagnosis and treatment of DKA.

## Introduction

Type 1 diabetes (T1D) can present with diabetic ketoacidosis (DKA) in 29.9% of cases, with a different prevalence across Europe (1). After a slight decrease in the frequency of DKA in Italy in children diagnosed with T1D under 15 years of age between 2014 and 2016 (2), recent studies showed that DKA risk, especially that of severe DKA, has significantly increased during the SARS-CoV-2 pandemic (3). According to the International Society for Pediatric and Adolescent Diabetes (ISPAD) Guidelines (4), hyperglycemia (>11 mmol/L, >200 mg/dl), acidosis (venous pH < 7.3 or serum bicarbonate <15 mmol/L), and ketosis (>3 mmol/L in blood) are necessary for a diagnosis of DKA. Higher values of glycemia (>600 mg/dl, >33.3 mmol/L), with no or mild acidosis (venous pH of >7.25, bicarbonate >15 mmol/L) and no ketonemia define the Hyperglycemic Hyperosmolar State (HHS) which can be observed, although rarely, in patients with T1D with consumption of high-carbohydrate beverages before diagnosis (5) or in young patients with other forms of diabetes (4). It is also possible to have a clinical presentation with mixed features of HHS and DKA, especially in those with severe dehydration with mild or moderate acidosis for other causes. Bicarbonate administration is not recommended except for the treatment of life-threatening hyperkalemia or unusually severe acidosis with evidence of compromised cardiac contractility (4).

We present three very similar case reports about adolescents with T1D at the onset with severe acidosis but low ketones to increase awareness of serum ketones (BOHB) monitoring.

**Table:** 

Nomenclature
T1D,	Type 1 Diabetes;
DKA,	diabetic ketoacidosis;
HHS,	Hyperglycemic Hyperosmolar State;
BOHB,	serum ketones;
BUN,	blood urea nitrogen;
AKI,	acute kidney injury;
ED,	Emergency Department;
PICU,	Pediatric Intensive Care Unit;
AG,	Anion Gap;
GCS,	Glasgow Coma Scale;
CT,	cranial tomography

## Case 1

A 40 kg 13- year-old female presented to be unresponsive in a peripheral Emergency Department (ED). She was unconscious, with a history of polydipsia and polyuria from a few days and vomit, rapid weak pulse, and deep labored breathing from the day before. Familiar and personal history was silent. Pediatric Glasgow Coma Scale (GCS) was 3, with arterial pressure of 90/60 mmHg. At venous blood gas analysis, a severe acidosis (pH 6.71, pCO_2_ 17.3 mmHg, HCO_3−_ 2.2 mmol/L) with hyperglycemia (550 mg/dl, 30.6 mmol/L) was evident. Hemoglobin was 13.4 g/dl, creatinine was 1.58 mg/dl (age normal values 0.4–0.7 mg/dl), and blood urea nitrogen (BUN) was 100 mg/dl (age normal values 6–20 mg/dl). Upon bladder catheterization, there were 1,000 ml of urine, of which analysis revealed glucose (3+), ketones (1+), and proteins (1+). A diagnosis of suspected DKA was performed and she received intubation and assisted ventilation followed by two boluses of crystalloid solution (normal saline 0.9%, 10 ml/kg each) through the intraosseous route. A saline solution at 5 ml/kg/h was commenced.

**Figure 1:**
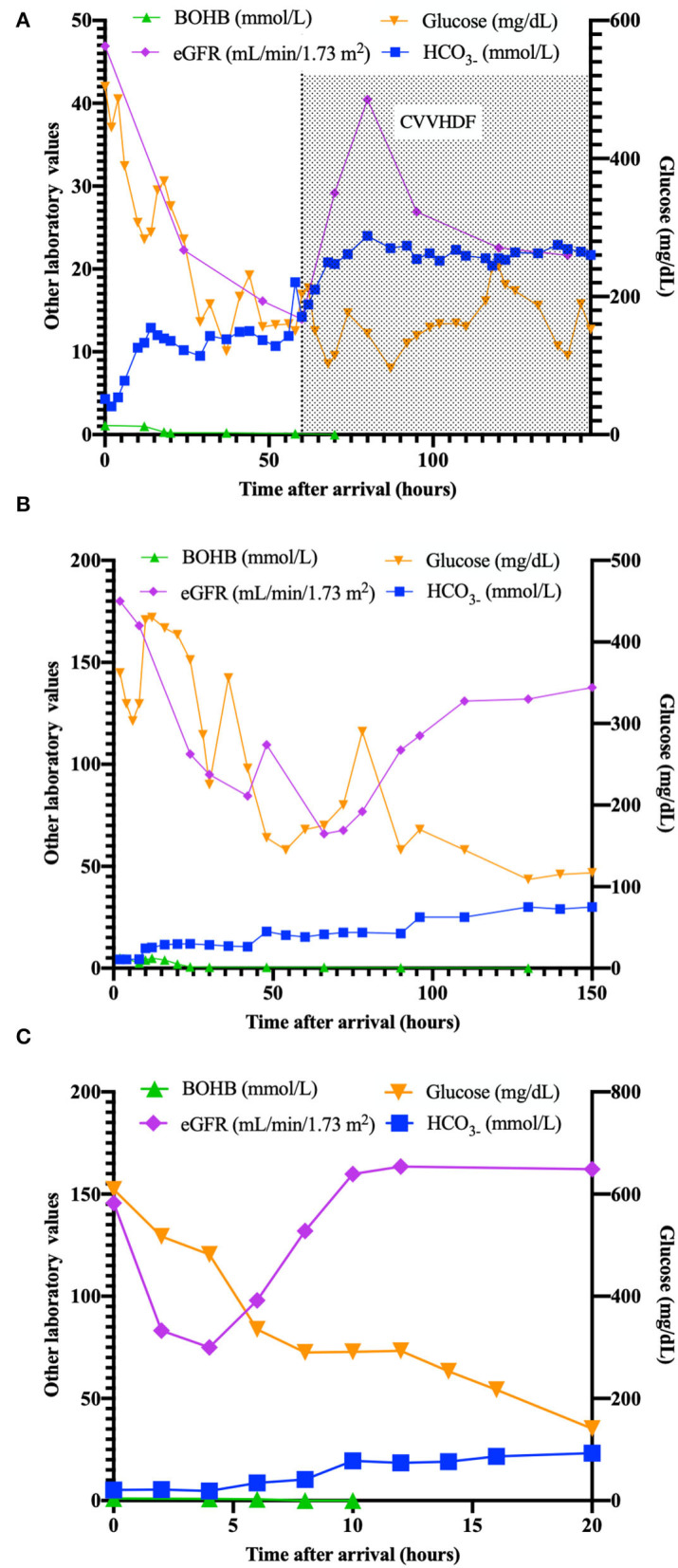
Plasma values (ketones, eGFR, bicarbonates, glucose) in patients **(A–C)** in the time after arrival (hours).

On arrival in our ED, she was still unresponsive, with acidosis (pH 6.775, HCO_3_- 4,3 mmol/L), Anion Gap (AG) of 22.7 mEq/L, persistent elevation of serum creatinine (1.2 mg/dl), and reduced estimated glomerular filtration rate (eGFR 48 ml/min/1.73 m^2^) with oliguria. Serum ketones were slightly increased (BOHB 1 mmol/L). A cranial tomography (CT) resulted to be normal. She was transferred to the Pediatric Intensive Care Unit (PICU) and received fluids, insulin (never exceeding 0.05 IU/kg/h), and potassium, but no bicarbonates, according to ISPAD Guidelines (4). Renal function progressively worsened, with increasing levels of creatinine (up to 4.21 mg/dl) with oligo-anuria despite the use of a diuretic (see Figure 1A). After 12 h, AG decreased to 7.1 mEq/L.

A diagnosis of acute kidney injury (AKI) (6) was made, and the persisting anuria required continuous venovenous hemodiafiltration. Ketones always returned negative (<0.1 mmol/L). T1D-specific antibodies resulted positive (IA2 1.01 UA/ml, GADA 43.6 UA/ml, ZnT8 105 UA/ml).

## Case 2

A 54 kg 10-year-old boy arrived in the ED with polyuria, polydipsia, and a weight loss of about 6 kg in 4 weeks. He also reported vomiting and dyspnea since the previous day. He showed poor general condition, dehydration, stable vital signs, heart rate of 131 beats/min, GCS of 15, and Kussmaul breathing.

At venous blood gas, a severe acidosis was present (pH 6.9, adjusted K+ 1.5 mmol/L, HCO3- 4.3 mmol/L, AG 22,2 mmol/L, glucose 362 mg/dl, SBE−27.8 mmol/L; ketonemia was 5.1 mmol/L).

The rehydration treatment started with 2.5 L/m^2^/24 h of saline supplemented with potassium (20 mEq/ L). After 2 h, insulin therapy in continuous intravenous infusion was added at 0.04 U/Kg/h, and gradually increased to 1.2 U/Kg/h. After the dropping of blood glucose <300 mg/dl, intravenous infusion was replaced with a solution made of 50% saline and 50% glucose-10% solution supplemented with potassium (40 mEq/L), without showing an improvement of pH and electrolytes at venous blood gas.

Rehydration and insulin therapy were continued for 8 h when the child worsened in his general condition, with a progressive reduction of GCS, persistence of dyselectrolytemia (especially hypokaliemia, with 1.6 mmol/L), and respiratory fatigue. Moreover, he presented an episode of desaturation and respiratory acidosis (pH 6.7, PCO2 100%), probably due to respiratory exhaustion despite a clear reduction in ketonemia (1.2 mmol/L) and a reduction of AG to 12 mmol/L. He was immediately transferred to the PICU where endotracheal intubation was performed and a brain CT scan was carried out, which resulted negative for cerebral edema.

While he was under ventilatory support, the child showed a gradual increase in BUN and creatinine values (about four times initial values), reduction of eGFR, persistence of dyselectrolytemia (K+ 1.6 mEq/L), with a diagnosis of AKI (6) (renal function and metabolic parameters are shown in Figure 1B). Venous blood gas showed an absence of ketonemia without an improvement of pH.

Bicarbonate intravenous infusion was then started with an initial dosage of 0.5 mEq/h which was progressively increased to 17.5 mEq/h (0.31 mEq/kg/h) with an improvement in pH value until complete resolution. Furosemide was also administered in order to react to diuresis contraction, with gradual improvement in creatinine levels. K-Cl supplementation was continued to resolve dyselectrolytemia. The next day, as conditions improved, it was possible to stop sedation and ventilatory support and gradually resume oral nutrition.

Bicarbonate intravenous infusion and furosemide helped us to resolve acidosis and prevent further kidney damage. After 48 h, insulin infusion was interrupted, and it was replaced with a multi-injection subcutaneous treatment. T1D-specific antibodies resulted positive (ICA 17.2 UA/ml, GADA 221 UA/ml).

## Case 3

An 31.8 kg 11-year-old boy arrived in the ED with the suspicion of T1D for the presence of polydipsia, polyuria, and weight loss (5 kg in 10 days) with obtundation and a tendency to sleep. Preceding his arrival to the ED, he received oral steroids and anti-histaminic drugs for urticaria, after which the child developed drowsiness.

On arrival in our ED, he had a modest general condition, with signs of dehydration, GCS 12, and accelerated breathing.

Medical history showed evidence of cardiopathy in the family (the mother was affected by Fallot tetralogy and retinal detachment). The child was otherwise healthy except for a cystic hygroma in the groin (diagnosed at the age of 3).

First blood gas analysis showed a pH of 7.07, pCO2 of 14.7 mmHg, Base Excess of−22.1, AG of 25.8 mEq/L, and HCO3- of 5.2 mmol/L with a glucose value of 609 mg/dl. Creatinine was 0.69 mg/dl (age normal values 0.4–0.7 mg/dl) with a K+ of 5.5 mEq/L and a sodium of 132 mEq/L. A normal saline solution was then commenced at 5 ml/kg/h according to guidelines (4). BOHB resulted to be modestly elevated (1 mmol/L).

After 2 h, values from blood gas analysis resulted comparable, with ketones slightly decreased (0.8 mmol/L), while AG resulted to still be elevated (24 mEq/L). Diuresis was reduced, with eGFR of 75 ml/min/1.73 m^2^, compatible with a diagnosis of AKI (6).

After nephrological consultation, a therapy with bicarbonate (0.375 mEq/kg/h), fluids (5 ml/kg/h), potassium (20 mEq/L), and insulin at 0.05 IU/kg/h was prescribed, with progressive resolution of acidosis and bicarbonate deficit. pH and bicarbonate returned to normal value after 10 h, while potassium normalized after 16 h. Diuresis returned normal (2.2 ml/kg/h) after 12 h (Figure 1C). T1D-specific antibodies resulted positive (GADA 1.64 UA/ml, ZnT8 37.1 UA/ml).

## Discussion

These are three similar case reports of adolescents at the onset of T1D with AKI, which commonly occurs in children with DKA (7). Despite severe acidosis and low bicarbonates, our patients had serum BOHB below 3 mmol/L, generally unassociated with DKA. In considering a DKA, it would always be important to take into account all differential diagnoses in children (lactic acidosis, metabolic acidosis, salicylate toxicity and septic shock). Also, the origin of metabolic acidosis could be different and/or seems to be very dynamic during the course of the condition.

These cases demonstrate that acidosis does not always arise from ketone-body accumulation in patients with hyperglycemia. In our cases, acidosis probably had a composite origin from defective bicarbonate reabsorption in the kidney and mild ketosis.

We hypothesized that massive polyuria induced severe dehydration with low volume and hypotension in association with metabolic decompensation that occurred in T1D at the onset. This situation might have initially led to hemodynamic damage with hypoperfusion and oligo-anuric AKI, followed by a more consistent ischemic tubular damage with the development of acute tubular necrosis. The altered tubular function did not allow bicarbonate reabsorption, and the buffering capacity was completely blunted, worsening underlying metabolic acidosis.

As previously reported (8), AG should be used to determine the moment of DKA resolution as opposed to using pH or bicarbonate. AKI can manifest with persisting non-AG acidosis, often self-limiting with conservative management. However, it would be important to intercept this complication early to avoid severe kidney damage.

In these presented cases, on arrival, AG were well above the upper limit (22.7, 22.2, and 25.8 mEq/L, respectively), which is suggestive for DKA. During the course of treatment, AG was not useful for all cases to differentiate AKI- from DKA-related acidosis, while serum ketone bodies were helpful early to intercept an acidosis unrelated to ketosis.

According to ISPAD guidelines (4), bicarbonate administration is not recommended except for treatment of life-threatening hyperkalemia or unusually severe acidosis with evidence of compromised cardiac contractility. This recommendation is due to possible side effects related to the infusion of bicarbonate, such as worsened hypokalemia, worsened intracellular acidosis due to increased carbon dioxide production, delay of ketoanion metabolism, and development of a paradoxical central nervous system acidosis.

However, in the second and third case, a rapid intervention in limiting the progression of AKI, with fluid infusion, better hemodynamic control, and through bicarbonate infusion, has helped to avoid a more consistent acute tubular damage as in the first case.

After the first hours, all patients demonstrated improvements that likewise happens for patients with classic T1D at the onset. All of them are now followed in the diabetes center of the hospital, and none have signs of organ damage (kidney, cardiovascular, brain) or worse glycemic control that could be related to the onset episode.

In conclusion, BOHB monitoring was helpful at first to rule out a suspicion of DKA and, in the following hours, was diriment to understand with great anticipation the acidosis origin and attribute it to tubular damage. We recommend serum BOHB measurement in every patient referring to ED with a suspicion of DKA to accordingly rule out other origins of acidosis and to treat patients.

In consideration of the increase in severe DKA cases reported during the pandemic (3), healthcare systems must be aware of the increase of possible DKA complications. In addition, pediatric emergency physicians must be prepared to manage very severe cases of DKA at the onset of T1D.

## Data Availability Statement

The raw data supporting the conclusions of this article will be made available by the authors, without undue reservation.

## Ethics Statement

Ethical review and approval was not required for the study on human participants in accordance with the local legislation and institutional requirements. Written informed consent to participate in this study was provided by the participants' legal guardian/next of kin. Written informed consent was obtained from the minor(s)' legal guardian/next of kin for the publication of any potentially identifiable images or data included in this article.

## Author Contributions

DT participated in the study design, writing the case series, and creating figures. SS and LP participated in the design of the study, collected data, and managed the literature search. LD helped to draft the manuscript. IR conceived the study and participated in its coordination, helped to draft, and review the manuscript. All authors contributed to the article and approved the submitted version.

## Conflict of Interest

The authors declare that the research was conducted in the absence of any commercial or financial relationships that could be construed as a potential conflict of interest.

## Publisher's Note

All claims expressed in this article are solely those of the authors and do not necessarily represent those of their affiliated organizations, or those of the publisher, the editors and the reviewers. Any product that may be evaluated in this article, or claim that may be made by its manufacturer, is not guaranteed or endorsed by the publisher.
